# Nutritional and Health Benefits of the Brown Seaweed *Himanthalia elongata*

**DOI:** 10.1007/s11130-023-01056-8

**Published:** 2023-03-22

**Authors:** Zahra Ilyas, Ali Ali Redha, Yuan Seng Wu, Fathima Zahraa Ozeer, Rotimi E. Aluko

**Affiliations:** 1 Department of Laboratory, Bahrain Specialist Hospital, P. O. Box: 10588, Juffair, Kingdom of Bahrain; 2 The Department of Public Health and Sport Sciences, Faculty of Health and Life Sciences, University of Exeter Medical School, University of Exeter, Exeter, EX1 2LU UK; 3 Centre for Nutrition and Food Sciences, Queensland Alliance for Agriculture and Food Innovation (QAAFI), The University of Queensland, Brisbane, QLD 4072 Australia; 4 Centre for Virus and Vaccine Research, School of Medical and Life Sciences, Sunway University, Selangor, 47500 Malaysia; 5 Department of Biological Sciences, School of Medical and Life Sciences, Sunway University, Selangor, 47500 Malaysia; 6 Department of Food and Human Nutritional Sciences, University of Manitoba, Winnipeg, MB R3T 2N2 Canada; 7 Richardson Centre for Food Technology and Research (RCFTR), 196, Innovation Drive, Winnipeg, MB R3T 2N2 Canada

**Keywords:** *Himanthalia elongata*, Seaweed, Bioactives, Brown algae, Marine phytochemicals, Nutraceuticals, Food ingredient

## Abstract

*Himanthalia elongata* is a brown seaweed containing several nutritional compounds and bioactive substances including antioxidants, dietary fibre, vitamins, fatty acids, amino acids, and macro- and trace- elements. A variety of bioactive compounds including phlorotannins, flavonoids, dietary fucoxanthin, hydroxybenzoic acid, hydroxycinnamic acid, polyphenols and carotenoids are also present in this seaweed. Multiple comparative studies were carried out between different seaweed species, wherein *H. elongata* was determined to exhibit high antioxidant capacity, total phenolic content, fucose content and potassium concentrations compared to other species. *H. elongata* extracts have also shown promising anti-hyperglycaemic and neuroprotective activities. *H. elongata* is being studied for its potential industrial food applications. In new meat product formulations, it lowered sodium content, improved phytochemical and fiber content in beef patties, improved properties of meat gel/emulsion systems, firmer and tougher with improved water and fat binding properties. This narrative review provides a comprehensive overview of the nutritional composition, bioactive properties, and food applications of *H. elongata*.

## Introduction

*Himanthalia elongata*, commonly known as Thongweed, sea thong or sea spaghetti, is an alga belonging to the order Fucales [1]. *H. elongata* is a cold-temperate fucoid commonly found in the Baltic, North Sea, and north-eastern Atlantic from Scandinavia to Portugal and Ireland. It lives on gently sloping rocky shores in low-lying and coastal zones, especially on shores with moderate wave loads. It is sometimes abundant and forms a distinct zone just below the Fucus serrated zone [2, 3]. *H. elongata* consists of small flat or discoid discs up to 3 cm wide with short stems. From autumn to winter, a long ribbon extends from the centre of the disc and branches several times. They have a rapid growth rate and can grow up to 2 m in height by the following summer with a disk life span of about 2–3 years [3].

The plant kingdom contains the best-studied families of naturally occurring antioxidants, phenolic chemicals, and carotenoid hues [4]. Although these useful additions can be obtained from sources other than land, plants in general and algae (seaweed) are great sources of natural antioxidants. Seaweeds flourish in harsh environments, releasing a wide range of antioxidant chemicals to combat environmental stressors [5]. Polyphenols, phlorotannin, flavonoids, carotenoids, polysaccharides, fatty acids, and amino acids are the most prevalent naturally occurring seaweed elements with antioxidant characteristics [6]. *H. elongata*, a brown maritime seaweed, is high in bioactive constituents [7] and, due to its antibacterial and antioxidant capabilities, plays a significant role in food production. [8].

From the literature research, it was found that no narrative review had been written on *H. elongata* up to date. Being an important source of bioactive compounds, this narrative review focuses on the presence of these compounds and their potential uses. Because of the presence of biologically active substances in algae, they play an essential role in therapeutic treatment; hence, pharmacological research is also included in this review. In addition, as a major source in the food industry, applications related to the food industry are discussed.

## Nutritional Composition of *H. elongata*

### Polysaccharides

Although brown algae are photosynthetic multicellular sea creatures, they have similarities to bacteria, mammals, plants, and even other algae in terms of their use of carbohydrates (alginates) [9, 10]. Fucales’ cell walls were chemically and enzymatically fractionated, and the results revealed that FCSPs and alginates were connected to various phenolic compounds while proteins and cellulose were tightly connected to FCSPs. The sulfated fucans from *H. elongata* had a consistent backbone structure of α-(1→3), but certain brown algae from Fucales had an alternating α-(1→3), (1→4) structure. Additionally, cellulose makes up just a small portion of the cell wall in brown algae (1–8% of algal dry weight), while sulfated fucans and alginates make up to 45% of the cell wall [9]. Similarly, it was determined that *H. elongata* have a very high fucose content with an amount of 26.3 g/kg [11].

The biopolymer specific to brown seaweeds such as *H. elongata* are alginates that are classified as polysaccharides. They have widespread biomedical purposes with minimal toxicity. A recent study used subcritical water extraction (SWE) on *H. elongata* in a pressurised reactor, with ensuing acetone fractionation to precipitate the crude fucoidan and liquid-phase containing alginate [10]. Calcium chloride was then added to the liquid phase to obtain calcium alginate precipitate that was further converted into sodium alginate. The yield of sodium alginate from SWE was 5.9% but increased to 15.9% with increasing acetone: hydrolysate volume ratios (0.5-2.0 v/v), indicating a greater yield than other Fucales species (10%). The corresponding sodium alginate products obtained from SWE and SWE with acetone fractionation (SWE_A) showed varying impact on viability (%) of T98G (Caucasian human glioblastoma), HCT-116 (colon carcinoma) and A549 (epithelial lung adenocarcinoma) cells. High cell viability was observed in HCT-116 with SWE, however in contrast, higher viability was observed in A549 and T98G cell lines with SWE_A.

### Dietary Fibre

Brown algae contain considerable amounts of dietary fibre [12] that contribute to a healthy gut and metabolic function. According to research, the total amount of dietary fiber found in *H. elongata*, collected from the coast of northwest Spain, was 37.14 ± 0.86% dry weight, of which soluble and insoluble dietary fiber made up 23.63 ± 0.48 and 13.51 ± 0.45% dry weight, respectively [13]. In this study, *H. elongata* had considerably more total dietary fibre (*P* < 0.05) than *Laminaria saccharina* (sweet kombu), *Mastocarpus stellatus*, and *Gigartina pistillata*.

It has also been investigated the *β*-D-mannuronic acid and *α*-L-guluronic acid ratio are present in edible seaweeds such *H. elongata.* [14]. The total dietary fibre, *β*-D-mannuronic acid and *α*-L-guluronic acid in canned *H. elongata* were determined to be 53.3 ± 3.5 (g/100 g dry weight), 78.2 ± 1.4 and 21.8 ± 1.4%, respectively, in comparison to dried *H. elongata* samples wherein the total dietary fibre was 42.7 ± 1.8 g/100 g dw, *β*-D-mannuronic acid was 78.3 ± 2.7% and *α*-L-guluronic acid was 21.7 ± 2.7%. The presence of these uronic acids has been shown to provide prevention against reactive oxygen species (ROS), thereby acting as reliable antioxidants [14].

### Amino Acids

It was found that *H. eloganta* had a total amino acid content of 54.02 ± 0.46 g/kg dry weight and contained high levels of lysine and methionine, which are essential for human nutrition [15]. A previous study reported a low protein content (6.8%) for the Spanish *H. elongata* [13].

### Fatty Acids

The fatty acid content of *H. elongata* collected from the Irish coast has been studied, it was found that in addition to 23.6% palmitic (C16:0), the algae produce high content of arachidonic acid (C20:4) (28.3%); 16.6% of stearidonic acid (C18:4), in addition to 10.7% *γ*-linolenic acid (C18:3), 10.6% oleic acid (C18:1), and 10.2% EPA (C20:5) [16]. Characterisation and analysis of Iberian coast’s *H. elongata* fatty acid content has reported (36.73 ± 2.16%) C16:0, (22.64 ± 1.80%) C18:1Ѡ9, (9.78 ± 2.27%) C20:4Ѡ6, and (2.77 ± 0.80%) C20:5Ѡ3 [17]. In a comparison study, two brown algae, *H. elongata* and *U. pinnatifida*, showed higher contents (0.79% and 7.87% dry matter, respectively) of polyunsaturated fatty acids (PUFAs) than the red algae *P. umbilicalis* [12].

### Sterols

Sterols, that are classified as lipids, have also been determined in *H. eloganta*. It was found that the predominant sterol was fucosterol measuring up to 2320 ± 187 *µ*g/g dw in canned *H. elongata* samples, and 1706 ± 150 µg/g dw in dried samples. Additionally, 24-ethylenecholesterol measured up to 2.6 ± 0.2% in canned *H. elongata* samples, and 2.6 ± 0.6% in dried samples [18]. The quantified amounts of macromolecules found in *H. elongata* has been reported in Table 1 with the respective analytical method.

**Table 1:** Quantified amounts of biomolecules and elements found in *Himanthalia elongata*

Biomolecules/elements	Amount	Sample Condition	Extraction method	Detection method	Reference
Amino acids	54.02 ± 0.46 g/kg DW	Dried sample	Dried and powdered. Acid hydrolysed	Ion chromatograhyby ninhydrin post-column reaction (PCR) technique	[15]
Macro-elements					
Sodium	25,805 ± 7924 mg/kg DW	Dried sample	Acid digestion and incineration in a muffle furnace	ICP-OES	[19]
Calcium	3469 ± 1526 mg/kg DW	Dried sample	Acid digestion and incineration in a muffle furnace	ICP-OES	[19]
Potassium	57,480 ± 19,976 mg/kg DW	Dried sample	Acid digestion and incineration in a muffle furnace	ICP-OES	[19]
Magnesium	3537 ± 1497 mg/kg DW	Dried sample	Acid digestion and incineration in a muffle furnace	ICP-OES	[19]
Trace Elements					
Boron	31.4 ± 16 mg/kg DW	Dried sample	Acid digestion and incineration in a muffle furnace	ICP-OES	[19]
Barium	3.39 ± 0.8 mg/kg DW	Dried sample	Acid digestion and incineration in a muffle furnace	ICP-OES	[19]
Cobalt	0.65 ± 0.14 mg/kg DW	Dried sample	Acid digestion and incineration in a muffle furnace	ICP-OES	[19]
Chromium	0.50 ± 0.70 mg/kg DW	Dried sample	Acid digestion and incineration in a muffle furnace	ICP-OES	[19]
Copper	2.2 ± 0.9 mg/kg DW	Dried sample	Acid digestion and incineration in a muffle furnace	ICP-OES	[19]
Iron	17.8 ± 3.3 mg/kg DW	Dried sample	Acid digestion and incineration in a muffle furnace	ICP-OES	[19]
Lithium	1.02 ± 0.6 mg/kg DW	Dried sample	Acid digestion and incineration in a muffle furnace	ICP-OES	[19]
Manganese	14.1 ± 12 mg/kg DW	Dried sample	Acid digestion and incineration in a muffle furnace	ICP-OES	[19]
Molybdenum	0.08 ± 0.03 mg/kg DW	Dried sample	Acid digestion and incineration in a muffle furnace	ICP-OES	[19]
Nickel	1.62 ± 0.2 mg/kg DW	Dried sample	Acid digestion and incineration in a muffle furnace	ICP-OES	[19]
Vanadium	1.82 ± 1.0 mg/kg DW	Dried sample	Acid digestion and incineration in a muffle furnace	ICP-OES	[19]
Zinc	21.3 ± 13 mg/kg DW	Dried sample	Acid digestion and incineration in a muffle furnace	ICP-OES	[19]
Sterols					
Fucosterol	2320 ± 187 µg/g DW	Canned	Saponification	HPLC-MS	[18]
	1706 ± 150 µg/g DW	Dried sample	Saponification	HPLC-MS	[18]
Dietary fibre					
Total dietary fibre	53.3 ± 3.5 g/100 g DW	Canned	AOAC gravimetric-enzymatic method	HPLC/LC-MS	[14]
	42.7 ± 1.8 g/100 g DW	Dried sample	AOAC gravimetric-enzymatic method	HPLC/LC-MS	[14]
*β*-D-mannuronic acid	78.2 ± 1.4% DW	Canned	AOAC gravimetric-enzymatic method	HPLC/LC-MS	[14]
	78.3 ± 2.7% DW	Dried sample	AOAC gravimetric-enzymatic method	HPLC/LC-MS	[14]
*α*-L-guluronic acid	21.8 ± 1.4% DW	Canned	AOAC gravimetric-enzymatic method	HPLC/LC-MS	[14]
	21.7 ± 2.7% DW	Dried sample	AOAC gravimetric-enzymatic method	HPLC/LC-MS	[14]
Vitamins					
Thiamine (B_1_)	0.26 ± 0.04 g/g DW	Canned	Acid and enzymatic hydrolysis	Reverse-phase HPLC	[20]
	0.14 ± 0.02 g/g DW	Dried sample	Acid and enzymatic hydrolysis	Reverse-phase HPLC	[20]
Riboflavin (B_2_)	0.31 ± 0.05 g/g DW	Canned	Acid and enzymatic hydrolysis	Reverse-phase HPLC	[20]
	1.14 ± 0.14 g/g DW	Dried sample	Acid and enzymatic hydrolysis	Reverse-phase HPLC	[20]
5-CH_3_–H_4_-folate	24.31 ± 0.83 µg/100 g DW	Canned	Heat treatment, deconjugation of folate polyglutamates using hog kidney conjugase, SPE and SPX purification	HPLC	[21]
	30.14 ± 4.85 µg/100 g	Dried sample	Heat treatment, deconjugation of folate polyglutamates using hog kidney conjugase, SPE and SPX purification.	HPLC	[21]
5-HCO–H_4_-folate	32.34 ± 3.57 µg/100 g DW	Canned	Heat treatment, deconjugation of folate polyglutamates using hog kidney conjugase, SPE and SPX purification.	HPLC	[21]
	46.96 ± 11.64 µg/100 g DW	Dried sample	Heat treatment, deconjugation of folate polyglutamates using hog kidney conjugase, SPE and SPX purification	HPLC	[21]
H_4_-folate	8.24 ± 0.83 µg/100 g DW	Canned	Heat treatment, deconjugation of folate polyglutamates using hog kidney conjugase, SPE and SPX purification	HPLC	[21]
	46.96 ± 11.64 µg/100 g DW	Dried sample	Heat treatment, deconjugation of folate polyglutamates using hog kidney conjugase, SPE and SPX purification	HPLC	[21]
Folic acid	17.59 ± 0.59 µg/100 g DW	Canned	Heat treatment, deconjugation of folate polyglutamates using hog kidney conjugase, SPE and SPX purification	HPLC	[21]
	25.81 ± 1.73 µg/100 g DW	Dried sample	Heat treatment, deconjugation of folate polyglutamates using hog kidney conjugase, SPE and SPX purification	HPLC	[21]
*α*-tocopherol	33.3 ± 4.2 µg/g DW	Dried sample	Pyrocatechol and KOH solution extraction	HPLC	[20]
	12.0 ± 2.0 µg/g DW	Canned	Pyrocatechol and KOH solution extraction	HPLC	[20]

Abbreviations: DW, dry weight; HPLC, high-performance liquid chromatography; HPLC-MS, high-performance liquid chromatography-mass spectrometry; ICP-OES, inductively coupled plasma optical emission spectrometry; LC-MS, liquid chromatography-mass spectrometry.

### Vitamins and Minerals

Water-soluble vitamins including thiamine (vitamin B1) and riboflavin are abundant in brown algae (vitamin B2). Flavin adenine dinucleotide and riboflavin mononucleotide, both of which are crucial for energy metabolism, are coenzyme [22]. Research was conducted to determine the concentration of thiamine and riboflavin from dry samples or canned sources of *H. elongata, L. ochroleuca, U. pinnatifida, Palmaria sp., and Porphyra sp*. [20]. It was determined that the thiamine content in dried *H. elongata* (0.14 *µ*g/g) and dried Porphyra (2.02 *µ*g/g) and the riboflavin content in canned *H. elongata* (0.31 *µ*g/g) and dried Porphyra (6.15 *µ*g/g) were calculated on a dry weight basis.

Folates are water-soluble natural form of vitamin B9 that may be found in a variety of foods. Depending on the degree of oxidation of the pteridine ring structure, foliates include a variety of compounds. Purine and pyrimidine synthesis as well as the synthesis of methionine from homocysteine both require vitamin cofactors [23]. Using HPLC, de Rodríguez-Bernaldo et al. [21] studied the content of folates in dehydrated and canned *H. elongata* along with other seaweeds. The folate was extracted through heat treatment, deconjugation and by purification methods. In dehydrated *H. elongata*, the concentrations of vitamers were found as: 5-CH_3_–H_4_-folate (30.14 ± 4.85 *µ*g/100 g dry weight), 5-HCO–H_4_-folate (46.96 ± 11.64 *µ*g/100 g dry weight), H_4_-folate (10.82 ± 2.96 *µ*g/100 g dry weight), and folic acid (25.81 ± 1.73 *µ*g/100 g dry weight). In comparison, the concentrations of vitamers in canned food were 5-CH_3_–H_4_-folate (24.31 ± 0.83 *µ*g/100 g dry weight), 5-HCO–H_4_-folate (32.34 ± 3.57 *µ*g/100 g dry weight), H_4_-folate (8.24 ± 0.83 *µ*g/100 g dry weight), and folic acid (17.59 ± 0.59 *µ*g/100 g dry weight) [21].

Seaweeds are rich in nutritional variables that are attracting an increasing interest, pertaining to their low-calorie content in addition to high levels of vitamins, minerals and dietary fibre. The presence of vitamin E, which is a generic name applied to tocopherols and tocotrienols in microalgae samples were confirmed by HPLC; an estimate of 33.3 ± 4.2 *µ*g/g dry mass of *α*-tocopherol was measured in dehydrated *H. elongata* and 12.0 ± 2.0 *µ*g/g dry mass found in canned *H. elongata* [24, 25].

Another research examined for trace elements (B, Ba, Co, Cr, Cu, Fe, Li, Mn, Mo, Ni, Sr, V, and Zn) as well as macro elements (Na, Ca, K, and Mg) *in H. elongata* and *Undaria pinnatifida*. When compared to *U. pinnatifida*, *H. elongata* exhibited the highest observed amounts of K (57480 mg/kg dry weight). However, it demonstrated relatively lower Fe concentration (58.8 mg/kg dry weight) [19]. In a comparative study, brown algae which are rich sources of K, Na, Ca, and Mg and have good Na/K ratios were reported to have significantly more minerals than red algae [26]. The quantified amounts of elements found in *H. elongata* are shown in Table 1 along with the respective analytical methods.

### Phytochemicals

Previous research has found that seaweed has an antioxidant capability that might be utilised to generate biopharmaceuticals with extensive medicinal uses [27]. Seaweeds are known as an important source of carotenoids [28], alginates [10] and phenolic compounds [29]. It has been demonstrated that brown algae contain more polyphenols than red and green algae. Phlorotannins, which have molecular weights ranging from 126 Da to 100 kDa and are structurally different polyphenols produced by the oligomerization and decoupling of the monomer phloroglucinol (1,3,5-trihydroxybenzene) [30, 31]. Phlorotannins are intricate polymers of the macroalgae compound phloroglucinol (1,3,5-trihydroxybenzene). The cell walls of brown algae are made up of these phenolic compounds. Additionally, they perform ecological tasks like UV resistance and grazing defense. To profile phlorotannin isomers in these macroalgae, phlorotannin fractions were increased using molecular weight cut-off dialysis and flash chromatography. Tests for antioxidant activity and total phenolic content are used as indicators. [32]. *H. elongata* also had considerably greater total phenolic content and antioxidant properties than nori (*Phorphyra*), kombu (*Laminaria*), and wakame (*Undaria*) [13]. The quantified amounts of phytochemicals found in *H. elongata* has been reported in Table 1 with the respective analytical method.

It was also clear that among other seaweed species and specific nutritional/bioactive components, *H. elongata* had the greatest total phenolic concentration (14.0 g/kg) [11]. To evaluate the quantitative and qualitative assessment of polyphenols in seaweeds, a recent study was undertaken on the optimization and validation of the reverse phase HPLC method [33]. Phlorotannins, hydroxybenzoic acid, hydroxycinnamic acid, and polyphenol flavonol subclasses are only a few of the seven phenolic chemicals that were found. The quantitative analysis of these compounds revealed the presence of 394.1 ± 4.33 *µ*g/g of phloroglucinol, 96.3 ± 3.12 *µ*g/g of gallic acid, 38.8 ± 1.94 *µ*g/g of chlorogenic acid, 44.4 ± 2.72 *µ*g/g of caffeic acid, 17.6 ± 0.85 *µ*g/g of ferulic acid, 8.6 ± 0.85 *µ*g/g of myricetin and 4.2 ± 0.15 *µ*g/g of quercetin in *H. elongata* extracted using 60% methanol extraction and cleaned with solid phase extraction.

Lipophilic compounds such as certain flavonoids and polyphenols as well as carotenoid pigments, flexibly act as primary or secondary antioxidants by obstructing hypervalent metals form generating and reacting with free radicals, as proven by several in vitro studies. The lipophilic compounds from three Irish brown seaweeds were also discovered for their antioxidant properties [34]. This study looked at the lipophilic antioxidants *of H. elongata*, *Laminaria saccharina*, and *Laminaria digitata*. Using an equal-volume mixture of organic solvents (chloroform, diethyl ether and n-hexane) for extraction, the highest total phenol (52.7 ± 1.93 to 180.2 ± 1.84 mg gallic acid equivalents/g), flavonoid (31.9 ± 2.65 to 131.3 ± 4.51 mg quercetin equivalents/g), carotenoid (2.19 ± 1.37 to 3.15 ± 0.91 *µ*g/g) and chlorophyll content (2.88 ± 1.08 to 3.86 ± 1.22 *µ*g/g) were obtained in the selected seaweed species. *H. elongata, L. saccharina*, and *L. digitata* lipophilic extracts showed significant antioxidant activity as well as the ability to chelate metal ions. In terms of antioxidant activity, *H. elongata* outperformed *L. saccharina, L. digitized*, and other species.

In a different study, TLC bioautography was used to extract several compounds from *H. elongata* in order to investigate their potential anti-inflammatory and antibacterial effects on Listeria monocytogenes bacterium. [28]. The isolated compound (fucoxanthin) shown high antioxidant (IC_50_: 14.8 ± 1.27 *μ*g/mL) and antibacterial action (inhibition zone of 10.27 mm at 25 g compound/disc). Fucoxanthin (Fx), a non-provitamin A carotenoid, is prevalent in brown algae and microalgae. It is known to attach to the chlorophyll a/c protein complex, which aids in photosynthetic organisms’ effective light gathering and body colour. Fx is thought to account for more than 10% of total body carotenoids [35].

## Nutraceutical Properties of *H. elongata*

The properties of brown algae inhabiting the north-western coast of the Iberian Peninsula reflect several health-promoting properties that may lead to their use in the food, pharmaceutical and cosmetic industries [36]. The concept of nutrients that consumers around the world benefit from has changed in recent years as consumers become more cautious towards more nutritionally healthy foods and their ingredients. The wide range of bioactive compounds mentioned earlier has given *H. elongata* a variety of nutraceutical properties that include: anti-mycotic, anti-histamine, anticholinergic, anti-photodamage, anti-osteoporosis, antioxidant, antidiabetic, hepatoprotective, anti-mycotic, anti-photodamage, anti-osteoporosis activities, as well as decreasing blood cholesterol, and preventing vascular thrombosis [37].

### Antioxidant Activity

According to epidemiological research, free radical production has a significant role in impacting human health via malignancies or age-related neurological illnesses [38]. However, advanced research has revealed that antioxidant-rich foods help to reduce damaging free radicals or ROS in the prevention of various diseases [39]. Previous research has shown that damaging free radicals, or ROS, play a crucial role in the etiology of chronic health issues such as cancer, cardiovascular disease, and neurological disorders [40]. Because of their high redox potential, the phytochemicals of *H. elongata* are regarded powerful antioxidants against ROS [33].

The presence of bioactive compounds in *H. elongata* and other brown algae has been widely studied and their concentrations were compared. Recent studies compared different brown algae and screened their bioactive properties followed by characterising antioxidant capacity [13, 19, 36]. Antioxidant treatment tends to minimize organic deterioration caused by excessive oxidative stress and can protect against the negative consequences of different traumas such as ischemia-reperfusion (I/R) [27]. One study showed the antioxidant capacity of *H. elongata* to protect against her I/R injury in the small intestine [41]. In this study, 72 male Wistar rats were randomly assigned to 12 different groups: sham, I/R only, I/R and vehicle at 3 time points, and I/R and extracted at 3 time points. The *H. elongata* extract-treated group showed significant differences (P < 0.05) in all parameters examined compared to the papillaless I/R group, thus *H. elongata* extract maintained normal enzyme levels. I can do it. Histological studies showed that intestinal mucosal damage was less severe in animals treated with *H. elongata* extract up to 24 h of reperfusion than in the untreated I/R group.

Fucoxanthin (Fx), an abundant compound in brown algae including *H. elongata*, along with fucoxanthinol (FxOH), the deacetylated type of Fx, to exert potential anticancer effects in preclinical cancer models through the suppression of many cancer-related signal pathways and the tumour microenvironment or modification of the gut microbiota [42]. In human and animal models, Fx has shown anti-inflammation [43, 44], anti-obesity [45], anti-diabetes [46], anti-hypertension, anti-cardiovascular disease [47], antimicrobial, antioxidant, photoprotective, anti-angiogenesis, anti-brain damage, and anticancer activities [48].

Colorectal cancer (CRC) is one of the ten most frequent cancers, although it is treatable with proper surgery and/or treatment. It is claimed that certain meals can help reduce the risk of CRC. Fx is recognized to reduce the risk of CRC due to its anti-cancer activity [42]. FxOH- and Fx-enriched algal extracts have been shown in human CRC cell lines, cancer stem cell-like spheroids, and CRC animal models to exhibit anticancer activity via different molecular pathways. It has been proposed that dietary and lifestyle changes can reduce the risk of CRC in people. As a result, intervention trials employing dietary or dietary-derived substances to study prevention have been conducted [42].

### Anti-hypoglycaemic Activity

Algae are also high in dietary fiber, which can help with glucose absorption and glycemic control. In a comparative study, *Porphyra umbilicalis, H. elongata*, and *U. pinnatifida* (Wakame) (Nori) were compared to investigate the *in vitro* inhibitory actions of various extract forms on glucosidase and glucose diffusion. *H. elongata* inhibited glucosidase activity significantly (*P* < 0.05), generating 26.2% lower glucose levels than controls. According to principal component analysis (PCA) done in this study discovered that soluble fiber and polyphenols were responsible for *H. elongata*’s enhanced nutraceutical activity. The *H. elongata* ethanol extract demonstrated the strongest inhibitory impact on glucose diffusion after 6 h (65.0 and 60.2%, respectively, vs. control). The extracts had the lowest slopes (68.2 and 62.8% vs. control, respectively) of the linear fits of glucose diffusion times. Algal effects on blood sugar have many different manifestations and are not always related. The studies to date suggest that *H. elongata* ethanolic and aqueous extracts could be helpful in creating functional meals [49].

Similarly, a prior research [50] investigated the *in vivo* hypoglycemic effect of different seaweed extracts in rabbits. Animals with normal blood sugar and triglyceridemia levels were used to study the effects of *H. elongata, Laminaria ochroleuca, Saccorhiza polyschides, Fucus vesiculosus*, and *Codium tomentosum* ethanol extracts. Eight hours after intravenous injection, *H. elongata* polysaccharides were found to significantly lower blood glucose levels. A 5 mg/kg crude polysaccharide dosage decreased blood glucose by approximately 18% in normal rabbits and 50% in alloxan diabetic animals.

### Neuroprotection Activity

The central nervous system (CNS) is significantly impacted by *H. elongata* as well. We investigated the analgesic, anticonvulsant, and muscle relaxing properties of a protein-rich algal solution. This includes impacts on body temperature, hyperactivity caused by amphetamine, exploratory behavior, and sleep caused by barbiturates. The extract was demonstrated to extend barbiturate-induced sleep. *H. elongata* can lessen CNS-related symptoms and possesses modest hypothermic, analgesic, and muscle relaxant [51]. In another research [52], found that *H. elongata* fraction (F1) changed analgesic activity, hyperlocomotion, motor coordination, rectal temperature, hypnosis brought on by pentobarbital, convulsions brought on by pentylenetetrazole, and analgesic activity using the writhing and hot plate tests. It was indicated that in the pentobarbital sodium-induced sleep test, F1 significantly reduced locomotor activity, hyperlocomotion, and rectal temperature while only slightly lengthening sleep time.

The two main sources of omega-3 fatty acids are phytoplanktons and seaweeds. One of the many omega-3 fatty acids, eicosapentaenoic acid (EPA), builds up in fish and in other marine animals that feed on algae and is then transferred to other species in the food chain. The CNS has been demonstrated to benefit from these fatty acids (FAs) throughout the growth of the fetal and new-born brain, retina, and nerve tissue. As a result, the importance of algae as a source of high-quality FAs for nutritional purposes is quickly growing, and it is vital to develop methods for maximizing extraction and evaluating their various levels in algae [53, 54].

## Applications in the Food Industry

The bioactive compounds such as phlorotannins, flavonoids, steroids, and sulfated polysaccharides of *H. elongata* may play important roles in food production due to their antibacterial and antioxidant properties. These secondary metabolites serve as potent defences against pathogens, inhibiting microbial growth and surviving under stressful conditions [55].

Fermentation of *H. elongata* was unsuccessful because neither heat-treated nor raw seaweed could support the growth of his *Lactobacillus plantarum*. However, its antibacterial activity against *Escherichia coli* and *Staphylococcus aureus* has been demonstrated [56]. The potent antibacterial activity of marine brown algae is attributed to phlorotannins. High concentrations of fucose and sulfate, and their placement in brown algae fibers, may contribute to this resistance to bacterial fermentation. Similarly, a study tested antimicrobial extracts from five food-approved species for efficacy against foodborne pathogenic bacteria *in vitro* (agar diffusion test) and *in situ* (microbial attack test) [60]. It was indicated that the extract with the highest phenolic content (18.79 ± 1.90 mg GAE/g) was obtained from *H. elongata.* The antibacterial effects also confirmed in food matrices may open up the prospect of their application as food preservatives [57].

It has been demonstrated that depending on the fatty acid concentration in *H. elongata*, the addition of 5% *H. elongata* alters the physicochemical, sensory, and microbiological characteristics of low-fat (10%) and PUFA-enriched frankfurters [58]. Seaweed significantly increased the hardness and crunchiness of PUFA-enriched low-fat frankfurters, decreased the brightness and redness, and enhanced the water-fat binding capacity (*P* < 0.05). The frankfurters with olive oil and seaweed had the highest total bacterial counts after 14 days of storage, indicating a lactobacillus-dominant microbiome as well as a successful food preservation technique.

Meat and meat products are an essential element of the daily diet, supplying essential nutrients (such as protein, iron, zinc, and B vitamins) for a healthy, balanced diet. The creation of functional meals based on meat can benefit from a variety of methods. The antioxidant capacity and composition of low-salt meat coupled with edible seaweed, for example, showed an increase (*P* < 0.05) in n-3 polyunsaturated fatty acids (PUFA) and a decrease (*P* < 0.05) in the *n*-6/*n*-3 PUFA ratio. *H. elongata* consumption resulted in a 20% increase in sulfur-containing amino acids in a low-salt meat composition. The addition of algae loaded the meat samples with soluble polyphenolic chemicals and boosted the system’s antioxidant capability. *H. elongata* samples had the highest increase in polyphenol content and antioxidants (*P* < 0.05) [59].

*H. elongata* is also thought to be a corroborate of antioxidants and nutritional fiber in beef patties. It enhances cooked patties’ physical, chemical, microbiological, and sensory qualities [60]. Beef patties cooked with seaweed were 50% more tender and had less cooking loss than those cooked without seaweed. Additionally, a significant rise in total phenolic content (up to 28.11 mg GAE/100 g body weight), dietary fiber (1.64 g/100 g body weight in a 40% seaweed pie), and DPPH radical scavenging activity (up to 52.32%) of patties with seaweed incorporation [60].

Similar to this, researchers looked at how *H. elongata* affected low-salt sausages made using konjac gel as a fat substitute in terms of emulsion stability, cooking loss, color, texture, residual nitrite, and microstructure [61]. Incorporating *H. elongata*/konjac gel caused a reduction in brightness and redness (*P* < 0.05) and an increase in yellowness as compared to other samples (*P* < 0.05). Depending on how much konjac gel is used in the formulation, seaweed has varying impacts on the textural qualities of reduced-salt frankfurters [61]. Frankfurter microstructure showed morphological alterations when the fat content was decreased and the konjac gel level increased. A more heterogeneous structure containing algae that were absorbed into the meat protein matrix was produced by the integration of *H. elongata*/konjac gel. [62].

Overall, *H. elongata* has demonstrated that its action essentially consists in reducing the presence of compounds that are detrimental to health and increasing the presence of beneficial compounds. The use of a meat-based functional diet (a mix of meat and 5% seaweed with or without dietary hypercholesterolemic agents) in developing animals (male Wistar rats) and their effects on different aspects of lipoprotein metabolism, oxidative stress and liver structure have demonstrated a significant role of seaweed in daily routine diet plan [63].

## Conclusions and Future Perspectives

This review article highlighted and summarized the quantified amounts of bioactive compounds found in *H. elongata* and their implications on various industries as previously reported in the literature. *H. elongata* is a brown seaweed that has been discovered to be a versatile nutraceutical over the years. It has demonstrated an excellent reference of phenolic compounds, antioxidants, fucose (26.3 g/kg) and potassium (57480 ± 19976 mg/kg) when compared to other seaweeds. With a total dietary fibre content of 53.3 ± 3.5 g/100 g dry weight under canned circumstances, *H. elongata* supports antioxidative activity by containing significant quantities of polyphenols, phlorotannin, flavonoids, carotenoids, fatty acids, polysaccharides, and amino acids. *H. elongata* demonstrated flexibility within the food industry wherein it has provided nutritional and texture enhancements for meat products such as frankfurters. In the pharmacological aspect, it has exhibited hypoglycaemic influence through inhibitory effects on *α*-glucosidase activity and on diffusion of glucose, demonstrated analgesic activity in the central nervous system and is the primary source for the biopolymer known as alginates (up to 15.9%), which is a non-toxic alternative for wound healing. *H. elongata* has also previously been discovered to mediate environmental toxicity caused by polycyclic aromatic hydrocarbons in the form of matrices. Through investigating a vast collection of research studies previously conducted on *H. elongata*, a clear significance on its role in the nutraceutical and pharmaceutical industry can be drawn. Finally, given the extensive diversity of applications of *H. elongata*, future research may evolve from *in vitro* testing to explore* in vivo* investigations.

## Data Availability

Not applicable.
